# Intricate Connection Among the Valsalva Maneuver, Gastrointestinal Tract, and Hemodynamics: A Rare Case Presentation

**DOI:** 10.7759/cureus.24474

**Published:** 2022-04-25

**Authors:** Christ Ordookhanian, Christine Barseghian, Ryan F Amidon, Paul Kaloostian

**Affiliations:** 1 Medicine, University of California, Riverside, USA; 2 Psychological Sciences, University of San Diego, San Diego, USA; 3 Medical Student, Medical College of Wisconsin, Milwaukee, USA; 4 Neurological Surgery, Riverside Community Hospital, Riverside, USA; 5 Neurological Surgery, Paul Kaloostian M.D. Inc., Riverside, USA

**Keywords:** hemodynamic response, mca stroke, migraine, arachnoid cyst, defecation, valsalva

## Abstract

A normal daily routine turns critical in seconds, and a biophysical maneuver that is instinctual leads to a rapid decline in a young healthy patient without any warning or precipitating signs. The Valsalva maneuver is a commonly used term for the act of bearing down that affects the vagus nerve, resulting in systemic changes primarily within the autonomic nervous system. This paper reviews a case in which a young man engaging in the Valsalva maneuver was later found unconscious and presented to the emergency department. Neuroimaging revealed a large middle cerebral artery stroke along with an undiagnosed temporal arachnoid cyst, without any significant historical medical records. The resulting course of this disease remained an interesting area of inquiry. This case highlights a rare but intricate interplay of several major physiological functions that collectively contribute to the unexpected demise of a young and healthy patient.

## Introduction

On a day-to-day basis, we repetitively go about our lives with minimal concern for the underlying network of organ systems working together in a tightly regulated and intricate manner. In this case, we aim to highlight one of the many complex physiological intricacies within the human body that often go unnoticed until pathology arises. When relatively healthy, the numerous processes that our bodies complete are not within our immediate conscious minds, such as the daily task of having a bowel movement. In this case, an otherwise healthy young male took a routine trip to the restroom, where an undiagnosed cyst adversely impacted normal mechanisms resulting in severe symptomology.

The gastrointestinal (GI) system starts at the mouth and ends at the anus, with the esophagus, stomach, and small and large intestines between each playing a vital role in the digestion of food and absorption of vital nutrients and water. In this case, we will focus on the physical manifestations of the Valsalva maneuver in defecation. Briefly, bearing down involves the physical action of inflating the lungs to peak tidal volume which is made possible by the contraction of the diaphragm, followed by the contraction of abdominal muscles, which ultimately raises intra-abdominal pressure. Muscular contraction and associated increased intra-abdominal pressure trigger peristalsis within the colon and rectum, allowing for the anal sphincter to relax and defecation to occur. All the while, there are systemic changes rapidly occurring to accommodate for such actions to occur. Of particular interest to this case is the hemodynamics during the muscular contraction phase where intra-abdominal pressure rapidly rises. When this occurs, systemic blood pressure transiently rises. The rise in intra-abdominal pressure also reduces venous return to the heart, effectively reducing the preload along with the overall cardiac output, causing a drop in systemic blood pressure and activation of baroreceptors within the carotid sinus and aortic arch which attempts to normalize systemic blood pressure. This process is manifested through the decreased influence of the vagus nerve, resulting in a transient increase in heart rate and restoration of blood pressure in healthy individuals. This process effectively describes the initial phase of the Valsalva maneuver.

Thus far, the superficially simple act of defecation has led us through a brief course in GI physiology, cardiovascular physiology, muscular physiology, and hemodynamics. How does this tie to the neurovascular system? The compensation for the decreased cardiac output that occurs in the later phase results in a sudden transient elevation of blood pressure. This results in an elevated intracranial pressure secondary to the rapid acceleration of blood into the brain. This burst of pressure within the neurovascular system is usually not of any notable concern in healthy individuals, but in this case, our patient had an undiagnosed arachnoid cyst, which typically presents with timely neurological symptoms or remains indolent until accidental discovery; rarely, do these malformations rupture. However, it is crucial to note that while arachnoid cysts are sacs filled with cerebrospinal fluid, they are also space-occupying and add to the overall pressure within the non-expandable enclosure of the cranium. Thus, when vagus nerve withdrawal causes transient tachycardia and elevated blood pressure, the resulting increase in rapid high-pressure shunting of blood toward the cerebral vasculature elevates the intracranial pressure, ultimately worsening the overall pressure within the cranium where an undiagnosed arachnoid cyst resides. As pressure builds within the cranium, the risk of serious pathology rises.

## Case presentation

**Figure 1:**
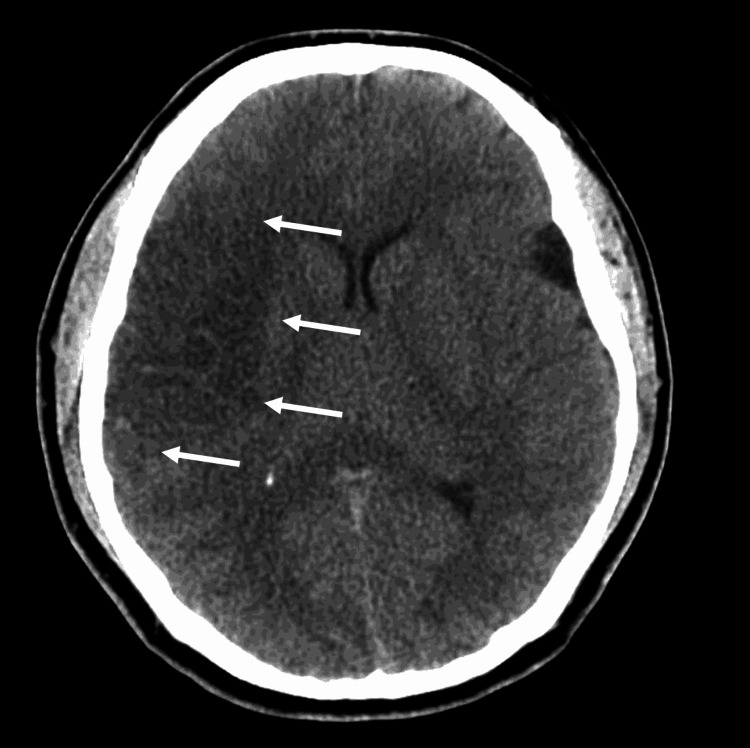
CT of the head revealed a significant right-sided MCA stroke (arrows) as well as a left-sided arachnoid cyst, further evaluated with MRI. CT: computed tomography; MCA: middle cerebral artery; MRI: magnetic resonance imaging

This interesting case involves a 28-year-old male who presented to the emergency department via emergency medical services after an unwitnessed loss of consciousness. Upon presentation, he was able to articulate the events preceding the collapse. Our patient stated that he had a history of migraines with a flare-up that occurred earlier that morning. He was bearing down for a bowel movement when he collapsed and was found by members of his household. Upon presentation to the emergency department, our patient was alert and orientated to self, time, and place and followed commands without difficulty. During the physical examination and review of systems, the patient examination was unremarkable except for left-sided hemiplegia. A computed tomography (CT) scan of the head revealed a right-sided middle cerebral artery (MCA) stroke and a left-sided arachnoid cyst in the temporal lobe (Figure 1).

**Figure 2:**
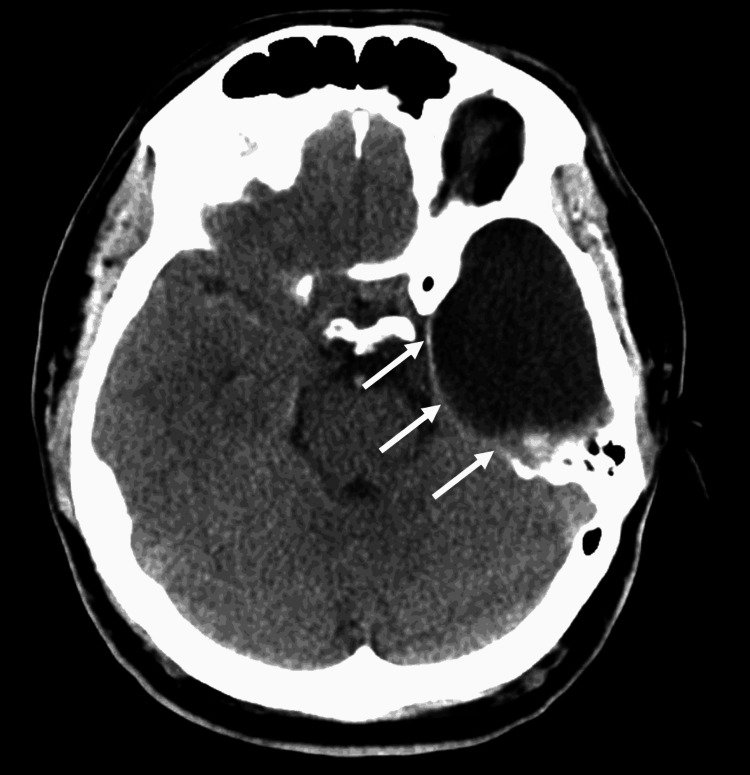
MRI of the head revealed a large left-sided arachnoid cyst (arrows) and redemonstrated the right-sided MCA stroke. MCA: middle cerebral artery; MRI: magnetic resonance imaging

The patient’s vitals and historical accuracy cleared all hurdles for tissue plasminogen activator (tPA) treatment, but he was not a prime candidate for thrombectomy given the large MCA stroke. A follow-up study with magnetic resonance imaging (MRI) confirmed the presence of a large right-sided MCA stroke as well as a large temporal arachnoid cyst on the left side (Figure 2).

Throughout hospitalization, several repeat CT scans were obtained. Both the imaging and the patient remained stable. Due to the size of the stroke and clinical presentation, the concern for subclinical increased intracranial pressure warranted treatment with 3% hypertonic saline and mannitol. A cardiology consultation was also acquired to screen for cardiogenic or vascular etiologies of our patient’s presentation. An echocardiogram was obtained with reassuring results to rule out immediate or pending cardiogenic etiologies (Table 1).

**Table 1:** Transthoracic echocardiography and transesophageal echocardiography reveal no abnormalities that could have contributed to the patient’s disease course. The left ventricular ejection fraction was within the reference range.

Transthoracic echocardiography	
Left ventricular ejection fraction	60-65%
Patent foramen ovale	No
Atrial septal defect	No
Wall motion abnormalities	No
Transesophageal echocardiography	
Bubble study	Negative

He had no family history of cardiovascular problems and no labs showing hypercoagulability. Despite our best medical intervention, his physical examination did not improve, but he was deemed a great candidate for rehabilitation therapy.

## Discussion

Common events that adversely influence defecation include constipation, diarrhea, and fecal incontinence. Constipation involves an abnormal reduction in defecation frequency and the potential for stool to harden. This combination is frequently associated with excessive straining when bearing down. Causes include a low-fiber diet, certain medications (particularly opioids), spinal cord injury, and multiple sclerosis [1,2]. Conversely, diarrhea involves an abnormal elevation in the frequency, volume, and/or liquidity of defecation, and fecal incontinence involves the lack of voluntary control over defecation [1].

Defecation is a four-step process involving the GI system, the nervous system, and the musculoskeletal system. When undigested dietary contents and waste reach the colon, they are shuffled into the rectum. Afferent nerves sense the resultant distention within the rectum via mechanoreceptors and stimulate the urge to defecate [2]. During this phase, the rectoanal inhibitory reflex occurs, characterized by involuntary relaxation of the internal anal sphincter to allow a minute portion of feces into the anal canal to sample its physical properties [2]. The expulsive phase includes actions to increase the pressure exerted on the anus by the rectum, reversing the rectoanal pressure gradient [2]. These include sitting/squatting, reflex relaxation of the internal anal sphincter and pelvic floor musculature, voluntary relaxation of the external anal sphincter, abdominal muscle contraction, and the Valsalva maneuver [1,2]. The end phase of defecation involves the closing reflex, restoring the tone of the anal sphincter and pelvic floor musculature, before returning to the basal phase.

In addition to facilitating the expulsive phase of defecation by stimulating peristalsis and reversing the rectoanal pressure gradient, the Valsalva maneuver affects the rest of the body as well. The technique begins with a deep inspiration by contracting the diaphragm, decreasing intrapleural pressure, and increasing venous return and intrathoracic blood volume, consequently reducing arterial blood pressure [3]. This activates baroreceptors within the carotid sinus and aortic arch, reducing the influence of the vagus nerve and triggering reflex tachycardia and peripheral vasoconstriction to restore the blood pressure. Additionally, as one exhales, straining the abdominal musculature, both intrathoracic and intra-abdominal pressures are elevated, resulting in a rise in arterial blood pressure [3]. The blood pressure correction and straining pressure result in a rapid, but limited, acceleration of blood into the neurovascular system, transiently elevating intracranial pressure while avoiding hyperperfusion [3].

Continued strain, and therefore increased intrathoracic and intra-abdominal pressure, ultimately reduces arterial blood pressure from the previously elevated position, with systolic pressure falling well below baseline, as cardiac output falls and peripheral venous pressure rises [3]. Abnormally high intrathoracic pressure generated while straining to expel difficult-to-move stool may significantly lower cerebral perfusion pressure at this point. Reflex tachycardia recovers the arterial blood pressure. When straining is discontinued and intrathoracic pressure returns to baseline, systemic arterial pressure rapidly drops before being corrected again by the sympathetic system [3]. While the hemodynamics of bearing down are typically uninteresting in healthy individuals, factors that already generate increased intracranial pressure at baseline create a risk for neurovascular compromise.

Arachnoid cysts are sheathed in arachnoid membrane and filled with cerebrospinal fluid, contributing to increased intracranial pressure as they occupy space within the cranium. Primary cysts are formed in utero, while secondary cysts may arise from mechanical stress, infection, or hemorrhage [4]. Typically discovered as an incidental finding, they are only found in about 1% of adults and are frequently asymptomatic [5]. The most common symptom caused by an arachnoid cyst mass effect is headache, although neurovascular compression can manifest with a plethora of symptoms [4].

In this case, hemodynamic changes during the strenuous act of bearing down during defecation led to an MCA stroke in a patient with a history of migraines who harbored an arachnoid cyst that elevated baseline intracranial pressure. The GI system may need to be acknowledged in patients with migraines and/or arachnoid cysts. Perhaps these patients should be counseled to avoid straining during bowel movements, use laxatives during instances of constipation, and promote gut health with adequate fiber, proper diet, and hydration.

## Conclusions

This case highlights a unique presentation of an underlying pathology that remained silent and out of clinical suspicion until a normal daily routine led to a significant complication, leading clinicians to formulate a differential diagnosis list that overlooked a simple physiological explanation for the precipitating events. We aim to highlight that in modern medicine with a vast array of tools available to our disposal, at times clinicians can overcomplicate disease processes and diagnostic criteria without considering basic physiological explanations for certain events. This case presents an interesting combination of bodily systems and their collaborative interactions that we often overlook in day-to-day life but can easily contribute to devastating pathology when homeostasis is disrupted. Our hope is to shed light on a unique case and foster a discussion among the medical community regarding a multisystem approach to preventative care in patients with pre-existing disease pathology.
